# Functional genomics identifies predictive markers and clinically actionable resistance mechanisms to CDK4/6 inhibition in bladder cancer

**DOI:** 10.1186/s13046-019-1322-9

**Published:** 2019-07-22

**Authors:** Zhichao Tong, Anuja Sathe, Benedikt Ebner, Pan Qi, Christian Veltkamp, Juergen E. Gschwend, Per Sonne Holm, Roman Nawroth

**Affiliations:** 1 Department of Urology, Klinikum rechts der Isar, Technical University of Munich, Ismaninger Strasse 22, 81675 München, Germany; 2 Center for Translational Cancer Research (TranslaTUM), Klinikum rechts der Isar, Technical University of Munich, Einsteinstrasse 25, 81675 Munich, Germany

**Keywords:** Bladder cancer, CDK4/6 inhibition, CRISPR, Resistance, Combination therapies

## Abstract

**Background:**

CDK4/6 inhibitors are a promising treatment strategy in tumor therapy but are hampered by resistance mechanisms. This study was performed to reveal predictive markers, mechanisms of resistance and to develop rational combination therapies for a personalized therapy approach in bladder cancer.

**Methods:**

A genome-scale CRISPR-dCas9 activation screen for resistance to the CDK4/6 inhibitor Palbociclib was performed in the bladder cancer derived cell line T24. sgRNA counts were analyzed using next generation sequencing and MAGeCK-VISPR. Significantly enriched sgRNAs were cloned and validated on a molecular and functional level for mediating resistance to Palbociclib treatment. Analysis was done in vitro and in vivo in the chorioallantois membrane model of the chicken embryo. Comparison of screen hits to signaling pathways and clinically relevant molecular alterations was performed using DAVID, Reactome, DGIdb and cBioPortal.

**Results:**

In the screen, 1024 sgRNAs encoding for 995 genes were significantly enriched indicative of mediators of resistance. 8 random sgRNAs were validated, revealing partial rescue to Palbociclib treatment. Within this gene panel, members of Receptor-Tyrosine Kinases, PI3K-Akt, Ras/MAPK, JAK/STAT or Wnt signaling pathways were identified. Combination of Palbociclib with inhibitors against these signaling pathways revealed beneficial effects in vitro and in in vivo xenografts.

**Conclusions:**

Identification of potential predictive markers, resistance mechanisms and rational combination therapies could be achieved by applying a CRISPR-dCas9 screening approach in bladder cancer.

**Electronic supplementary material:**

The online version of this article (10.1186/s13046-019-1322-9) contains supplementary material, which is available to authorized users.

## Background

There were an estimated 549,393 newly diagnosed cases of bladder cancer and 199,922 deaths in 2018 worldwide [1]. Despite advances in diagnosis and treatment, incidence rates and mortality have not significantly changed over the past decades [2–4]. Recently, a novel class of small molecules targeting cyclin-dependent kinase 4/6 (CDK4/6) including Palbociclib, Abemaciclib and Ribociclib have been approved by the FDA for treatment of hormone receptor positive breast cancer in combination with hormone therapy [5, 6]. Around 93% of bladder cancers (BLCA) display mutations in genes regulating cell cycle progression. We and others have demonstrated the efficacy of CDK4/6 inhibition in pre-clinical studies of BLCA [7–10]. However, a multicenter phase II trial evaluating Palbociclib monotherapy in patients with metastatic urothelial carcinoma after failure of first-line chemotherapy (NCT02334527) was terminated as the defined endpoint was not met [11, 12]. In clinical trials, monotherapy with CDK4/6 inhibitors has failed to provide long-term anti-tumor effects in almost all tumor entities examined, mostly due to de novo and acquired resistance mechanisms [12–15]. In preclinical studies, predictive markers have been identified by application of diverse experimental approaches. Expression of RB1 has been demonstrated to be an essential marker for response to CDK4/6 inhibitors but there are also a few reports that observed RB1 independent sensitivity [10, 16]. Molecules involved in the regulation of RB1 function such as CDKN2A, CDKN2B, CCND1, p16INK4A and MDM2 have been associated with response to CDK4/6 inhibitors [16]. Transient inhibition of pRb with CDK4/6 inhibitors has been demonstrated to be compensated by CDK2 [16, 17]. These data support the general importance of the RB1 signaling network in the response to CDK4/6 inhibitors. Activation of signaling pathways such as the PI3K/AKT, MAPK or JAK/STAT pathway also confer resistance to treatment and represent potential predictive biomarkers [18, 19]. Combination therapy strategies targeting these pathways with CDK4/6 inhibitors are synergistic and are being investigated in early phase multi-agent clinical trials in various tumor types [20, 21]. These recent developments indicate that combination therapies are a powerful strategy to improve responses to CDK 4/6 inhibition. However, identification of the most effective combination therapies together with the identification of genomic aberrations as predictive biomarkers of response is now of utmost importance.

One strategy to gain understanding of molecular mechanisms underlying the response to CDK4/6 inhibitors is to interrogate gene function by manipulating gene expression level. For this purpose, we used a genome-scale gain of function (GOF) screen with a CRISPR-dCas9 synergistic activation mediator (SAM) system in conjunction with a sgRNA library containing 70,290 sgRNAs against 23,430 genes [22–24]. This method has previously been successfully used to identify predictive markers and molecular mechanisms of resistance to a BRAF inhibitor in melanoma [24].

We describe here the identification of resistance mechanisms to CDK4/6 inhibition in BLCA by applying this GOF CRIPSR-dCas9 screen approach. From these results, we devised rational combination therapies that overcame diverse resistance mechanisms.

## Materials and methods

### Cell culture

T24 (ATCC® HTB-4™), MCF7 (ATCC® HTB-22™) and HEK293T (ATCC® CRL-11268™) cell lines were obtained from the American Type Culture Collection (ATCC, Manassas, VA, USA) with passage number 47, 60 and 14, RT112 (DSMZ no. ACC 418) was obtained from the Leibniz institute German collection of microorganisms and cell cultures (Braunschweig, Germany). RT112-Luciferase cell line was a gift from Dr. Per Sonne Holm, Department of Urology, TUM. All cell lines were maintained in DMEM or RPMI medium (Biochrom AG, Berlin, Germany) in 10% or 5% CO2 respectively, supplemented with 10% FBS, 1% P&S and 1% NEAA (Biochrom,AG,Berlin, Germany). Bladder cancer cell lines T24 and RT112 were further verified with genotyping (Additional file 6: Table S1a).

### Small molecule inhibitors

Stock solutions of Everolimus, Ruxolitinib, Stattic, SH-4-54, Erdafitinib, Axitinib, CI-1040, Roscovitine (Selleckchem, Munich, Germany), MK-2206 (Active Biochem, Bonn, Germany), PIK-90 (Merck Chemicals GmbH, Darmstadt, Germany) were prepared in DMSO while NVP-BEZ235 was in DMF. Palbociclib (Sigma-Aldrich Chemie GmbH, Munich, Germany) stock solution was prepared in water. Working concentrations were freshly prepared in medium.

### Cloning, library amplification, and DNA production

Human CRISPR 3-plasmid activation pooled library (SAM) was a gift from Feng Zhang (Addgene #61426) [24]. SAM library amplification was carried out as described using Endura electrocompetent cells (Lucigen Corporation, Middleton, WI, USA) and BioRad Genepulser II (Bio-Rad Laboratories GmbH, Munich, Germany). DNA was prepared using Endofree Maxi or mini kits (Qiagen, Hilden, Germany) following the manufacturer’s protocol.

### Production of lentivirus

Lentivirus was produced as described earlier [7, 25]. 2 million 293 T cells were seeded on 10 cm plates and transfected with 10 μg DNA, 15 μg of psPASX2 and 6 μg pMD2.G using 2.5 M CaCl2 and 2x HBS. Medium was changed 6 h after transfection and virus supernatant was collected after 48 h.

### Functional titration of lentivirus

Different volumes of viral supernatant were applied to T24 cells in a 6 well format and treated with selection antibiotics for 72 h. Cell viability was determined by SRB assay, and the multiplicity of infection (MOI) for the virus was calculated as described [26].

### Generation of T24 SAM cells with lentivirus transduction

T24 cells were transduced with lentivirus containing dCas9-VP64 and MS2-p65-HSF1 [24] respectively. Antibiotics (blasticidin, hygromycin B from Life Technologies) for selection pressure were applied to the cells 24 h after transduction and selection pressure was continued for 8 days. Monoclonal colonies were isolated and expression of dCas9-VP64 and MS2-p65-HSF1 were measured.

### Palbociclib resistance screen

T24 SAM cells transduced with 0.2 MOI of lentivirus containing the sgRNA library were used for the screen. 35 million cells were transduced to maintain a 500-fold representation of the 70,290 gRNAs. After 8 days of Zeocin (300 μg/ml, Life Technologies) selection, cells were used as control or treated with Palbociclib. Treatment was conducted with 1000 nM Palbociclib daily for 7 days. Virus production and resistance screen were conducted as three independent replicates.

### Genomic DNA extraction and next-generation sequencing (NGS)

Genomic DNA was extracted from cells using an ammonium acetate-based method as described previously [27]. To control for representation, PCR with gRNAs sequence primer listed in Additional file 6: Table S1b was performed on genomic DNA equivalent to 500 cells per guide, corresponding to a total of 231 μg from 35 million cells (assuming 6.6 pg in a single diploid cell). This input DNA was split into 77 reactions with 3 μg per reaction. For the verification of amplified SAM gRNA library, 10 ng of input plasmid DNA was used. PCR was conducted using Phusion Flash High-Fidelity PCR master mix (Life Technologies) for 28 cycles as 98 °C for 90 s, 98 °C for 1 s, 60 °C for 5 s, 72 °C for 15 s, followed by final extension of 72 °C for 1 min. To enable multiplexing during NGS, an 8 bp unique barcode was added at the beginning of the forward primer as TCGCCTTG, ATAGCGTC, GAAGAAGT, ATTCTAGG or CGTTACCA. All PCR products were pooled, ethanol precipitated and resuspended in 100 ul. This was subjected to electrophoresis on a 2% agarose gel and the band of interest was extracted using Monarch DNA Gel extraction kit (NEB GmbH, Frankfurt, Germany) according to manufacturer’s instructions. NGS libraries were prepared from amplicons by GATC Biotech (Konstanz, Germany) and 125 bp paired end sequencing was carried out on an Illumina HiSeq 4000 with 15 million reads per condition.

### Analysis of NGS data

FastQC was used for quality control of NGS data at the sequence level [28]. NGS reads were demultiplexed according to the 8 bp barcode in the forward primer using cutadapt [29]. Adapter and primer sequences were trimmed using cutadapt to yield 20 bp gRNA sequences, with no mismatches permitted for both steps. Screen hit identification, quality control and visualization were performed using MAGeCK-VISPR (version 0.5.7) [30, 31]. ‘count’ and ‘test’ commands from MAGeCK were used with default parameters and zero count removal. A list of human housekeeping genes were applied as biologically negative control [32]. MAGeCK results were then used as input for VISPR.

### Gene set enrichment analysis

Pathway analysis of candidate genes was performed using DAVID V 6.8, Reactome Version 65 [33, 34].

### CRISPR/Cas9 synergistic activation mediator (SAM) for individual gene targeting

*sgRNAs and validated non-target control sgRNA* [35] were cloned *into lenti-sgRNA (MS2)-zeo-backbone (addgene #61427)* as described [36] and verified by Sanger sequencing. T24 SAM cells were transduced with lentivirus at MOI 0.05 and selected for zeocin resistance (300 μg/ml). SgRNA expression and mRNA expression fold change were detected with qPCR as described in resulting polyclones (sgRNA sequences and primers are listed in Additional file 7: Table S2) [9].

### Cell viability assay and cell proliferation assay

Cell Titer-Blue cell viability assay (Promega G8081) and Sulforhodamine B (SRB, Sigma-Aldric S7635-100MG) cell proliferation assay were conducted according to the manufacturer’s protocol after exposure to inhibitors for indicated time periods and daily medium change. For the SRB assays, the cells were fixed, stained and rinsed with 1% acetic acid. Then 10 mM Tris base solution (pH 10.5) was applied and the absorbance was measured at 510 nm. These assays were conducted in 12 or 24 well dishes by seeding 500 or 1000 cells. Cells were incubated with control or drug for 3 or 7 days for understanding short or long term effects respectively. The incubation time for Cell Titer-Blue reagent was 4 h at 37 degrees Celsius.

### Caspase-Glo 3/7 assay (apoptosis assay)

Caspase-Glo® 3/7 (PromegaG8091) assay was conducted according to manufacturer’s protocol and normalized to a parallel Cell Titer-Blue assay.

### Cell cycle analysis

Cells were harvested and fixed in 75% cold ethanol overnight, washed with 1% BSA/PBS and stained using 4 μg/mL 7-Aminoactinomycin D (7-AAD, Thermo Fisher Scientific) according to the manufacturer’s instructions. Flow cytometry was performed using FACSCanto II flow cytometer (BD Biosciences) and analyzed with FlowJo v.7.6.4 software (Tree Star, Inc., Ashland, OR).

### Clonogenic assay

Cells were seeded in 6-well plates at a density of 30 cells per well and treated for 7 days with Palbociclib 1000 nM. Medium containing inhibitor was replaced every second day. Colonies were fixed and stained (6% v/v glutaraldehyde and 0.5% w/v crystal violet) as described [37] and imaged on a Zeiss Axio Vert.A1 microscope. Colonies that consisted of more than 50 cells were counted.

### Chicken chorioallantoic membrane (CAM) assay

The CAM assay was performed as described previously [38]. In brief, 2 million RT112 Luc cells were seeded on ED (Embryo Day) 9 and topically treated with respective inhibitors on ED 11–14. After adding D-Luciferin potassium salt (Sigma), luminescence intensity was measured on ED 15 using the HAMAMATSU Digital Camera C9016 (Hamamatsu Photonics K.K.) and quantified with Simple PCI (Imaging Systems, Compix Inc. Cranberry Township, PA, USA).

### Immunoblotting and antibodies

Immunoblotting was conducted as described previously using ECL™ Prime Western Blotting System (GE Healthcare) [9]. The primary antibodies used included: Akt (pan) (C67E7) Rabbit mAb #4691, Phospho-Akt (Thr308) (C31E5E) Rabbit mAb #2965, Phospho-p70 S6 Kinase (Thr389) (108D2) Rabbit mAb #9234, p70 S6 Kinase Antibody #9202, Phospho-p44/42 MAPK (Erk1/2) (Thr202/Tyr204) (20G11) Rabbit mAb #4376, p44/42 MAPK (Erk1/2) Antibody #9102, Phospho-Rb (Ser780) (D59B7) Rabbit mAb #8180,GAPDH (14C10) Rabbit mAb #2118, Cyclin D1 (92G2) Rabbit mAb #2978, CDK2 (78B2) Rabbit mAb #2546, Phospho-CDK2 (Thr160) Antibody #2561, Keratin 7 (D1E4) Rabbit mAb #4465 from Cell Signaling Technology, Purified Mouse Anti-Human Retinoblastoma Protein Clone G3–245 (RUO) #554136(BD Pharmingen™), Recombinant Anti-Uroplakin Ia antibody [EPR15498] (ab185970) and Recombinant Anti-NuMA antibody [EP3976] (ab109262) from Abcam, Anti-Cas9, clone 7A9 (Cat.#MAC133) from EMD Millipore Corporation. Secondary HRPO conjugated antibodies were purchased from Dianova.

### Combination index analysis

The dose-inhibitory fraction relationships for the combination therapy were assessed with the Chou–Talalay combination index (CI) analysis [39]. Additive effects were defined as 0.9–1.1, CI < 0.9 as synergistic and CI > 1.1 as antagonistic. The analysis was performed with CompuSyn (Combo Syn, Inc., Paramus, NJ, USA).

### Statistical analysis

Statistical analyses were performed using GraphPad Prism (version 6.0) software (GraphPad PrismSoftware, Inc). Data were analyzed for statistical significance using unpaired t-test/one-way ANOVA. For all experiments, *p* < 0.05 was considered statistically significant.

## Results

### Identification of sgRNAs that confer resistance to CDK4/6 inhibition

**Fig. 1:**
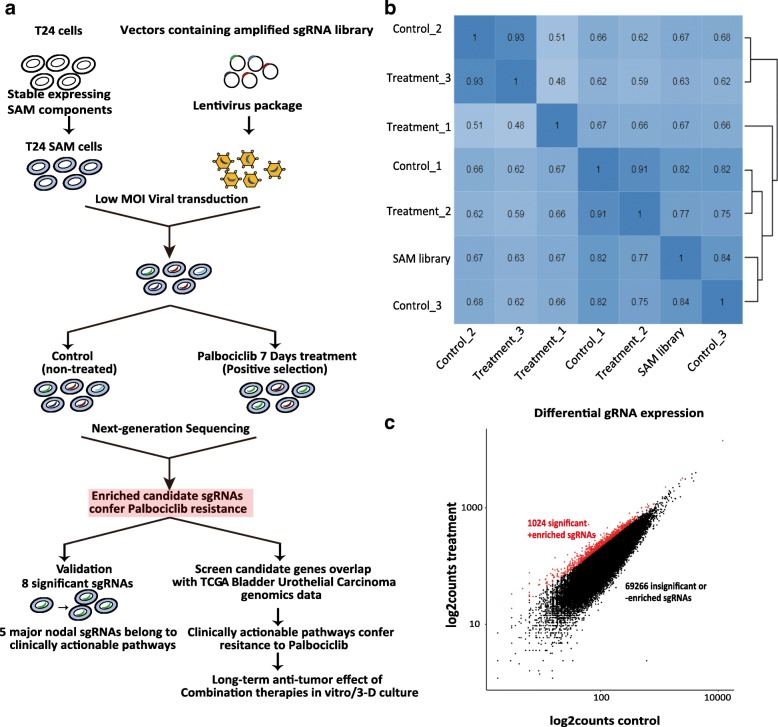
Identification of sgRNA candidates that confer resistance to Palbociclib. **a** Schematic overview of functional screening for candidate genes that confer resistance to CDK4/6 inhibition and translational workflow. **b** Clustering and Pearson correlation of NGS results of all biological replicates. “SAM library” indicates the amplified SAM library while 1, 2, 3 indicate independent bio-replicates of control and treatment with Palbociclib. **c** Individual gRNA counts were plotted for control and Palbociclib treatment conditions. Red dots indicate counts that are significantly enriched in Palbociclib treatment according to MAGeCK analysis, after applying cutoffs of *p* < 0.1 (FDR corrected) and LFC > 0.25

In order to determine resistance mechanisms to CDK4/6 inhibition, we applied a genome-scale transcriptional activation screen using a CRISPR-dCas9 based system including a synergistic activation mediator (SAM) and a pool of 70290 sgRNAs as described schematically in Fig. 1a [24]. We decided to use the bladder cancer derived cell line T24 for this screen since it is representative of genetic alterations found in a large proportion of muscle invasive bladder cancers [40]. T24 cells were transduced with the dCas9-VP64 fusion protein and the MS2-p65-HSF1 activation helper and expression was confirmed by immunoblots and PCR (Additional file 1: Figure S1a). Monoclone #2 referred as T24 SAM resembled parental T24 cells in morphology, growth characteristics (data not shown), expression of urinary protein markers and response to Palbociclib (Additional file 1: Figure S1b,c) and was used for conducting the screen. Viral multiplicity of infection was calculated by functional titration of the produced lentivirus (Additional file 1: Figure S1d). Genomic DNA was isolated and sgRNA read counts were analyzed using next-generation sequencing. All conditions displayed a similar sequence quality score from 30 to 40 (error probability 0.001–0.0001), approximate mapped reads, Gini index and read counts (< 0.1) without skews (Additional file 1: Figure S1e,f,g,h). Comparison across the independent replicates revealed between 0.06–0.5% zero counts. In detail, 76 sgRNAs were lost by amplification of the sgRNA library but still 100% of the RefSeq coding isoforms were covered. We lost additional 72–253 sgRNAs across the bio-replicates of control and 82–355 in the treatment group indicating that experimental conditions were adequate to maintain the sgRNA library representation (Additional file 1: Figure S1i).

Comparison of the read counts from control replicates and sgRNA library demonstrated a Pearson correlation coefficient value of 0.67–0.84 and between control and treatment replicates the value was 0.51–0.93. Three biological treatment replicates demonstrated a Pearson correlation coefficient value of 0.48–0.59 comparable to the original publication for this library (Fig. 1b) [24, 31]. The MaGeCK software was used to compare differential expression of individual sgRNA with the treatment and control replicates and identified 1024 candidate sgRNAs that were positively enriched with a FDR cutoff < 0.1 plus LFC (log2 fold change) > 0.25 in the treated cells (Fig. 1c, Additional file 7: Table S2.1). Hence, genes encoded by these enriched sgRNAs enable cell proliferation in the presence of Palbociclib and can be regarded as mediators of resistance.

### Validation of sgRNA candidates

**Fig. 2:**
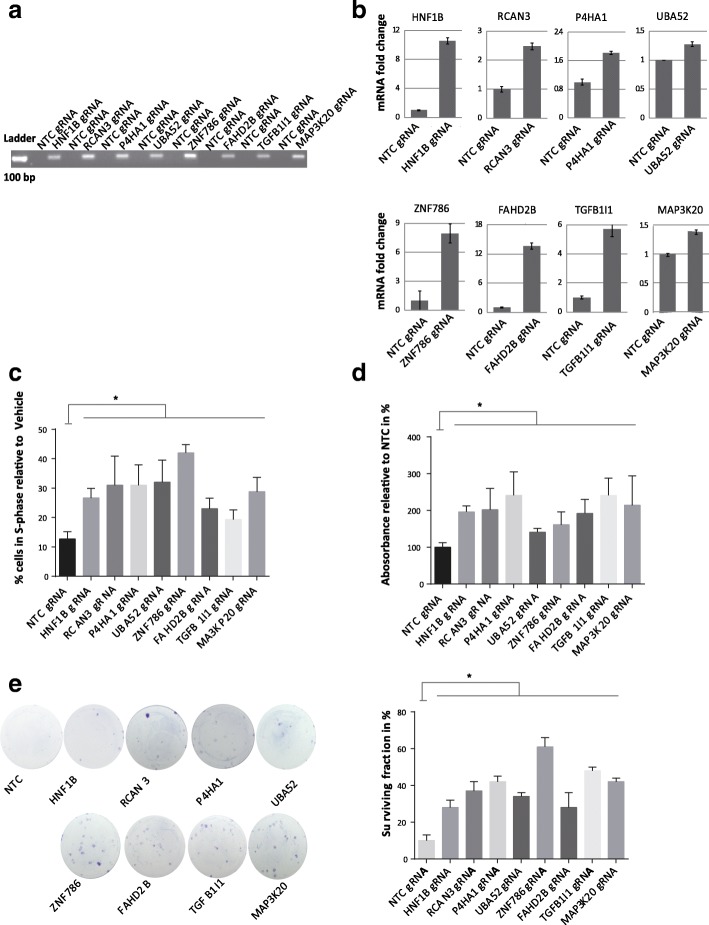
Validation of significant sgRNA candidates (**a**) Expression of selected sgRNA transcript sequences in engineered T24 SAM cells was analyzed by PCR using primer listed in Additional file 6: Table S1b. **b** Detection of mRNA expression level of the 8 candidate genes relative to the NTC (**c**) Percentage of cells in S-phase 24 h after Palbociclib (1000 nM) treatment normalized to vehicle-treated control (*, *P* < 0.05; unpaired t-test compared percentage of cells in S-phase under Palbociclib treatment of cells to NTC). **d** SRB cell proliferation assay after 4 days of Palbociclib (1000 nM) treatment of cells transduced with sgRNA candidates normalized to NTC (*, P < 0.05; unpaired t-test comparing normalized absorbance of cells treated with Palbociclib or NTC). **e** Images and quantification of colonies of a 10-day clonogenic assay under Palbociclib (1000 nM) treatment. (Surviving fraction = colony number/seeded cell number in %; * means P < 0.05; unpaired t-test). Data represent the mean ± SD of 3 replicates

We performed biological validation for 8 of the sgRNAs that were ranked within the first five hundred most significantly enriched sgRNAs using a random number generator (Table 1). The respective target proteins were located in the PAK pathway, TGF-β pathway, GPCR pathway, collagen chain trimerization, RNA Polymerase II Transcription Initiation and Promoter Clearance and Wnt signaling pathway. The sgRNAs were cloned into T24 SAM cells and examined for their effect on Palbociclib treatment. Expression of all sgRNA transcripts (Fig. 2a) and a 1.3 to 13-fold increase in the mRNA transcription level of the target genes as compared to non-target control sgRNA transduced cells (referred to as NTC) was observed (Fig. 2b). All 8 sgRNAs expressing cells had increase in proportion of cells proceeding into S-Phase after Palbociclib treatment (30–350%) as compared to NTC cells (Fig. 2c). Significant increase in cell viability from 41 to 141% was noted 72 h after treatment in cells expressing target sgRNAs (Fig. 2d). We also examined the effect of sgRNAs on survival and proliferation under long-term Palbociclib treatment using clonogenic assays conducted over 7 days. All sgRNAs tested induced increased number of colonies compared to NTC (Fig. 2e). This indicates that all tested sgRNA confer partial resistance to treatment and that the genes encoded by these sgRNAs might serve as predictive marker for therapy response.

**Table 1:** sgRNAs chosen for biological validation

sgRNA	Gene	LFC	FDR	Rank
NM_000458_1073	HNF1B	3.0416	4.39E-69	1
NM_013441_42667	RCAN3	2.9917	1.57E-21	3
NM_001142595_12383	P4HA1	1.954	2.21E-08	13
NM_001033930_5799	UBA52	1.4143	2.60E-08	15
NM_152411_62833	ZNF786	1.2864	0.004702	252
NM_199336_69028	FAHD2B	1.8803	0.006676	306
NM_001042454_6659	TGFB1I1	1.1487	0.01617	425
NM_016653_47440	MAP 3 K20	1.1754	0.023672	499

### Activation of multiple signaling pathways confer resistance to Palbociclib

**Fig. 3:**
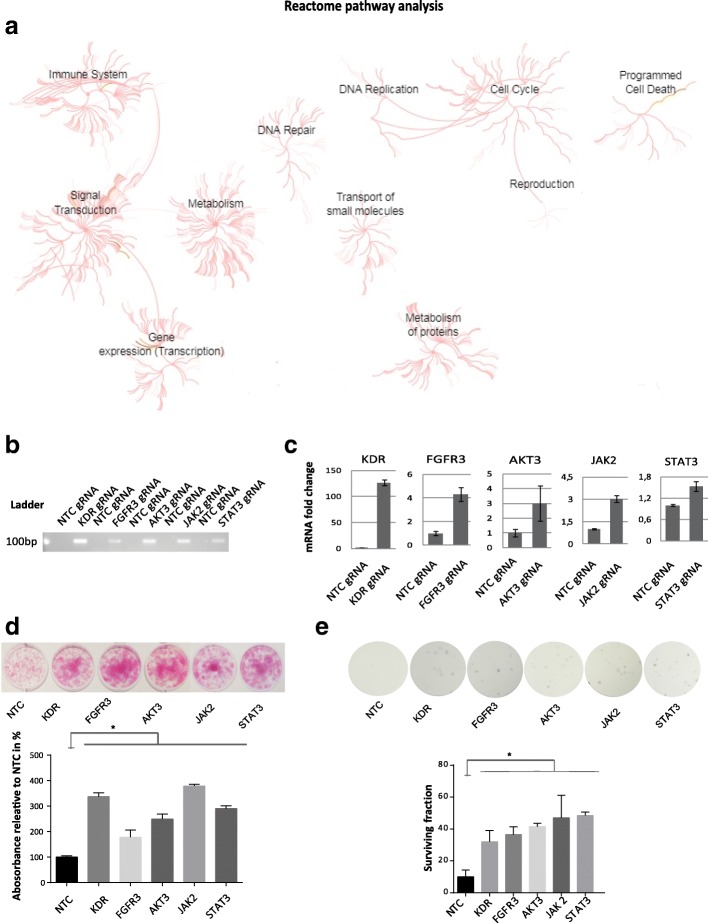
Identification and validation of oncogenic signaling pathways that confer resistance to Palbociclib treatment. **a** Reactome pathway analysis with sgRNA candidates that confer resistance to Palbociclib. **b,c** Transcriptional activation of the 5 candidate genes was confirmed by qPCR in T24 SAM cells transduced with the according sgRNAs. **d** Detection of cell growth using a SRB cell proliferation assay after 4 days of Palbociclib (1000 Nm) treatment. Bar graph indicates the absorbance relative to NTC (*, P < 0.05; unpaired t-test), (**e**) 10 day clonogenic assay under Palbociclib (1000 nM) treatment. (Surviving fraction = colony number/seeded cell number in %; * means P < 0.05; unpaired t-test). Data represent the mean ± SD of 3 replicates

For analyzing the biological and clinical significance of the candidate sgRNAs, all enriched 1024 sgRNAs from the screen were translated into official gene symbols using DAVID, resulting into 995 candidate genes (Additional file 7: Table S2.2). To link those potential predictive marker of resistance with clinical data, we compared the candidate genes with genetic data from comprehensive analysis of muscle-invasive bladder cancers characterized by multiple TCGA analytical platforms on cBioPortal [41]. We included only candidate genes with amplification in copy number alteration (CNA) assuming this should indicate gene activation, resulting in a remaining 882 genes (Additional file 7: Table S2.3) [41–43]. Analysis of integrative pathways with these genes was performed using Reactome pathway analysis, that identified distinct signaling pathways including cell cycle, DNA repair, programmed cell death, metabolism or signal transduction (Fig. 3a). Targeting functional units of those pathways might overcome therapy resistance to monotherapy with Palbociclib. We also performed KEGG pathway analysis using DAVID, in which ‘metabolism’ and ‘pathways in cancer’ were most significantly overlapped with our candidate genes. Signaling pathways included in the term ‘pathways in cancer’ were analyzed for the availability of clinically applicable drugs using the database DGIdb [44], which identified several Receptor Tyrosine Kinases and the PI3K-AKT, Ras/MAPK, cell cycle and JAK-STAT pathway (Table 2). To validate hyperactivation of those pathways with therapy resistance, the genes *KDR, FGFR3, AKT3, JAK2, STAT3* were selected because they are frequently amplified in bladder cancer [45]. sgRNAs targeting above genes were transduced into T24 SAM cells and therapy response to Palbociclib was examined. Expression of all 5 sgRNAs and increase in the transcriptional level of target genes was confirmed (Fig. 3b,c). The sgRNA transduced cells acquired partial resistance against Palbociclib, as examined by cell viability and clonogenic assays conducted over 10 days when compared to NTC cells (Fig. 3d,e).

**Table 2:** Inhibitors/agonists for combination therapy with Palbociclib

Inhibitors/agonists	Target	Pathway
PIK-90	PI3Kα/γ/δ	PI3K-AKT
MK2206	Akt1/2/3	PI3K-AKT
Everolimus	mTOR	PI3K-AKT
NVP-BEZ235	p110α/γ/δ/β and mTOR	PI3K-AKT
Axitinib	VEGFR1/2/3, PDGFRβ and c-Kit	Ras/MAPK
Erdafitinib	FGFR1/2/3/4	Ras/MAPK
CI-1040	MEK1/2	Ras/MAPK
Ruxolitinib	JAK1/2	JAK-STAT
Stattic	STAT3	JAK-STAT
SH-4-54	STAT3/5	JAK-STAT
Roscovitine	CDK2/7/9	Cell cycle

### Validation of synergistic combination therapies

Above results suggested that amplification of distinct pathways in bladder cancer can serve as indicators of resistance to Palbociclib. In order to target the oncogenic signaling pathways identified and validated in Fig. 3b-e, we analyzed DGIdb (?) for inhibitors against these pathways that are in clinical development. This led to the development of a panel of 11 different inhibitors that were tested as monotherapy and combination therapy with Palbociclib in T24 and RT112 cells (Table 2). We included RT112 cells in our validation studies as they possess molecular alterations frequently described in bladder cancer but distinct to T24 cells [41, 46]. Dose-response kinetics of these inhibitors/agonists and Palbociclib were performed in vitro using cell viability assays.

Non-linear regression dose-response curves were performed based on above cell viability data to determine the IC50 for combination therapies (Additional file 2: Figure S2a,b). With the Chou-Talalay method the combination index (CI) was calculated that revealed synergism for Palbociclib in combination with Axitinib, Erdafitinib, CI-1040, NVP-BEZ235, PIK90, MK2206, Roscovitine and Everolimus (Table 3, Additional file 8: Table S3). However, inhibitors against the JAK/STAT signaling pathway showed additive and even antagonistic effects.

**Table 3:** IC50 and Combination Index analysis of combination therapies on T24

	IC50 (95% CI,nM)	Effect (IC50 + Palbociclib 1000 nM)	Combination Index
Axitinib	919.1 (754.8–1119)	0.63	0.67
Erdafitinib	3752 (3296–4271)	0.79	0.43
NVP-BEZ235	9.12 (6.755–12.31)	0.67	0.33
CI-1040	1661 (1225–2254)	0.87	0.2
MK2206	1260 (407.6–3897)	0.55	0.44
PIK90	1581 (908.4–2753)	0.74	0.16
Everolimus	3.501 (1.605–7.637)	0.65	0.15
Roscovitine	4815 (3094–7492)	0.81	0.72
SH-4-54	2324 (2129–2537)	0.57	2.93
Stattic	2384 (2015–2820)	0.53	0.88
Ruxolitinib	21221 (18381–24500)	0.54	3.85

**Fig. 4:**
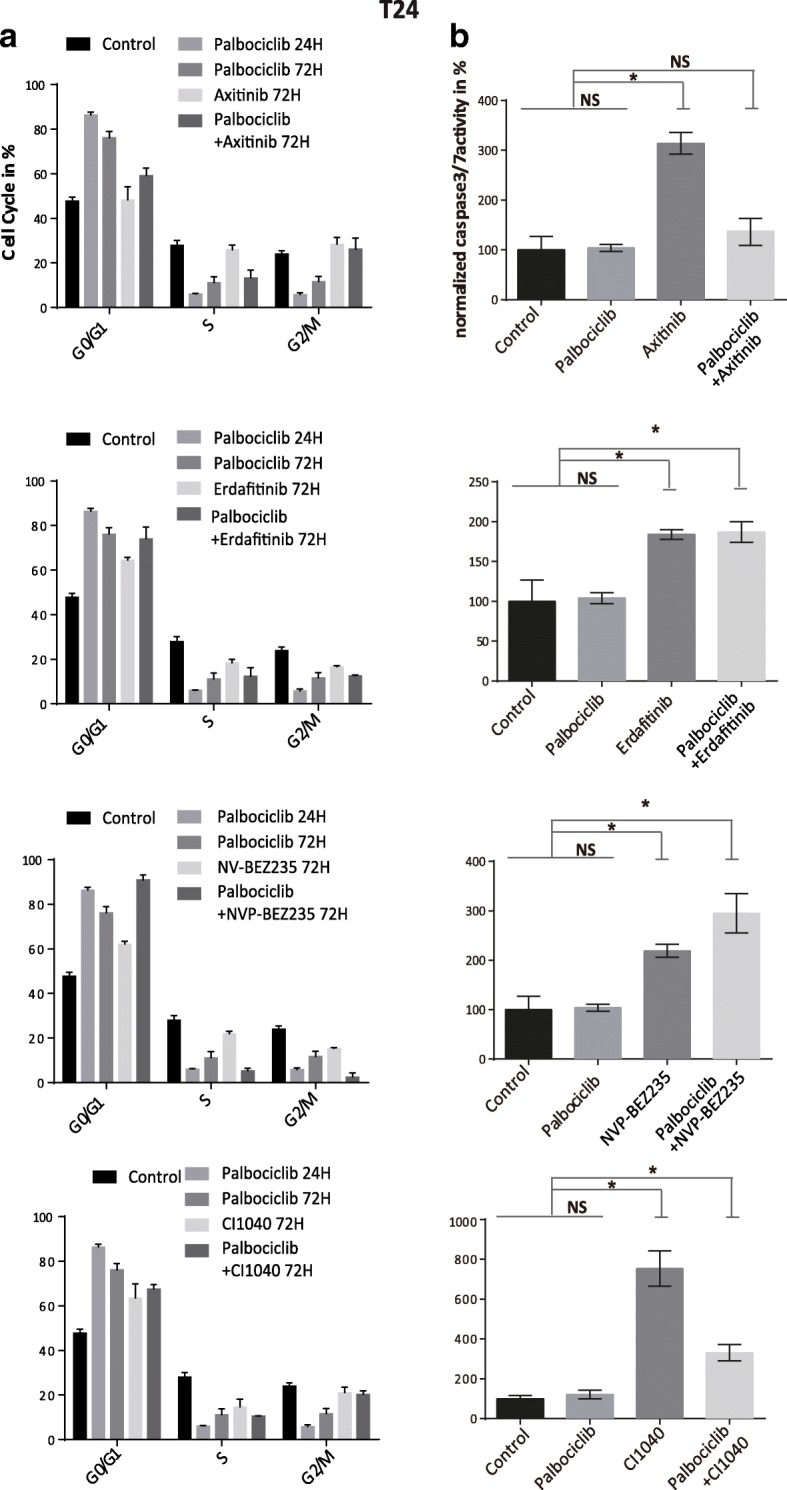
Anaylsis of cell cycle progression and Caspase3/7 activity in T24 cells with mono- or combination therapies (**a**) Analysis of cell-cycle progression of T24 cells 72 h after mono- or combination therapies as depicted (Data represent the mean ± SD of 3 replicates). **b** Caspase3/7 activity was measured and normalized to CTB results after 72 h of treatment (*, P < 0.05; one-way ANOVA with Dunnett’s multiple comparisons test, and unpaired t-test; NS not significant, Data represent mean ± SD of 3 replicates). Concentrations used were Palbociclib (1000 nM), Axitinib (1000 nM), Erdafitinib (5000 nM), NVP-BEZ235 (200 nM) and CI1040 (1000 nM)

Since treatment of cells with Palbociclib mostly arrests bladder cancer cells in G0/G1 cell cycle stage by inhibiting progression into S-Phase, we examined if the synergistic drug combinations with Axitinib, Erdafitinib, CI1040 and the PI3K/mTOR inhibitor NVP-BEZ235 could induce an even greater G0/G1 arrest than monotherapy. Palbociclib monotherapy almost completely abolished progression into S-Phase in T24 and RT112 cells in the first 24 h, but after 72 h a partial recovery was detectable (Fig. 4a, Additional file 3: Figure S3a). When using combination therapies, the effect at 72 h differed between the two cell lines examined. In T24 cells only the combination with NVP-BEZ235 suppressed cell-cycle progression with only 5.3% of cells in S-phase compared to 11.2% in Palbociclib monotherapy, whereas in RT112 cells the combination with Axitinib, Erdafitinib or NVP-BEZ235 inhibited cell-cycle progression to 3.9, 3.7 and 2.88% in S phase compared to Palbociclib monotherapy (8.5%). We also measured apoptosis defined by caspase-3/7 activity at 72 h after treatment, which was not induced by Palbociclib monotherapy. In contrast, all 4 compounds tested induced significant caspase 3/7 activation in mono- and combination-therapy on both cell lines. However, in T24 but not RT112 cells, the combination of Palbociclib with Axitinib or CI-1040 reduced activation of caspase 3/7 compared to monotherapy without Palbociclib (Fig. 4b, Additional file 3: Figure S3b).

We also analyzed expression and phosphorylation status of key proteins in the targeted pathways in both cell lines. Palbociclib monotherapy resulted in a downregulation of p-RB and p-CDK2 24 h after treatment followed by a partial recovery at 72 h. Hyperactivation of AKT and ERK could be observed in all conditions. This hyperactivation could not be suppressed by the PI3K/mTOR inhibitor NVP-BEZ235 or MEK1/2 inhibitor CI-1040 in combination with Palbociclib. But still in T24 cells all combination therapies could suppress phospho-RB recovery partially, while the recovery was completely blocked in RT112 cells. We also noticed that the mTOR downstream target S6K1 got hyperphosphorylated on both T24 and RT112 cells with Palbociclib monotherapy at 72 h. But only in T24 cells this effect could be suppressed with the NVP-BEZ235 combination therapy (Additional file 4: Figure S4,Additional file 5: Figure S5 a,b,c,d). We conclude that although the mechanism of action is mediated via both, cell cycle progression and apoptosis, contribution of these two parameters on the therapy response can be differently regulated dependent on the genetic background of these cell lines.

### Combination therapies induce long term effects on tumor growth

**Fig. 5:**
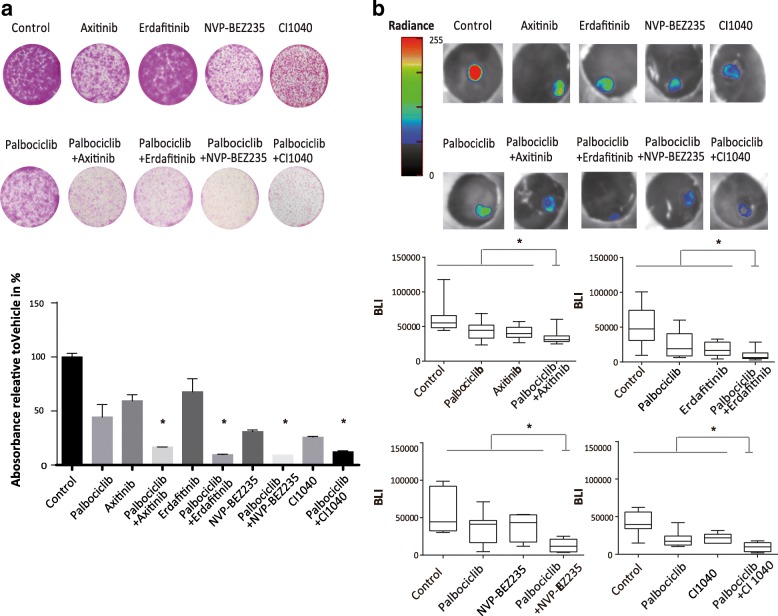
Detection of long-term synergism in vitro and in vivo. **a** Analysis of cell proliferation using a SRB assay 7 days after treatment with Palbociclib (1000 nM) or in combination with Axitinib (1000 nM), Erdafitinib (5000 nM), NVP-BEZ235 (200 nM) and CI1040 (1000 nM) (*, P < 0.05; one-way ANOVA with Dunnett’s multiple comparisons test, combination therapies compared to respective monotherapies and non-treated control, data represent the mean ± SD of 3 replicates). **b** RT112-Luc xenografts were treated as described and size was measured by quantification of the luminescence signal, representing the number of viable cells (*, P < 0.05; one-way ANOVA with Dunnett’s multiple comparisons test, combination therapies compared to respective monotherapies and non-treated control)

The long-term effects of these 4 combination therapies was further evaluated by using a 7-day SRB proliferation assay. In both, T24 and RT112 cells the combination of Palbociclib with either NVP-BEZ235, Erdafitinib, Axitinib or CI-1040 were synergistic (Fig. 5a and Additional file 3: Figure S3c). To further evaluate the efficacy of combination therapies in a three-dimensional tumor xenograft system, we used a RT112-luc xenograft model on the chicken chorioallantoic membrane (CAM) model [38]. Response of RT112-luc cells to Palbociclib treatment resembled RT112 parental cells (data not shown). All xenografts were randomized to non-treated control and treatment with Palbociclib, Erdafitinib, Axitinib, CI-1040 or NVP-BEZ235 as mono- or combination therapy. The combination therapies induced a statistically significant synergistic antitumor effect compared with monotherapies without increasing mortality of the developing chicken embryo (Fig. 5b).

## Discussion

Identification of molecular mechanisms of resistance to target therapies is a prerequisite for improving treatment efficacy by designing rational combination therapies. We applied a combination of a forward genetic screen using CRISPR-dCas9 technology and the analysis of large-scale cancer genomics data sets from patients to identify molecules and signaling pathways that confer resistance to the CDK4/6 inhibitor Palbociclib in bladder cancer. This strategy allowed us to identify not only molecules that are involved in therapy response to CDK4/6 monotherapy but also revealed a network of frequently mutated signaling pathways in patients that confer resistance.

For minimizing off-target effects induced by the sgRNAs design or genomic integration, the SAM library was designed with minimal predicted off-target activity [22, 47] and we used low-MOIs for transduction. The Pearson correlation coefficient between biological treatment replicates in our analysis was comparable to other CRISPR screen approaches that have a range from 0.25–0.92 overall [41, 48]. However, reproducibility among replicates remains a subject for improvement. We used one of the earliest systems developed but novel CRISPR based sgRNA libraries and expression systems have been developed recently that might overcome those potential limitations [49, 50].

Validation of single sgRNAs and also validation of signaling pathways activated by the screen candidate sgRNAs revealed a range of single molecules and different pathways with diverse biologic functions involved in resistance mechanisms. We could demonstrate that overexpression of single genes by using random sgRNAs identified in the screen did result in partial resistance to Palbociclib treatment. Also, besides already described signaling pathways involved in the acquired resistance to CDK4/6 inhibitors, the sgRNA panel identified in the screen resulted in modeling a network of signaling pathways that influence therapy response to CDK4/6 inhibitors that extended current knowledge such as involvement of “programmed cell death”, “metabolism” or “transport of small molecules”. Development of novel inhibitors might allow improvement of combination therapies with CDK4/6 inhibitors by characterizing those pathways in greater detail. The diversity of different signaling events involved in the cellular response to CDK4/6 inhibitors reflect probably the diverse and complicated downstream signaling network of CDK4/6 as described only recently [20, 51, 52]. This diversity of molecular candidates that confer resistance might also explain why the identification of single reliable predictive markers was not successful in clinical trials to date. It also supports the notion that the response to CDK4/6 inhibitors is complex and cannot be reduced to a single predictive marker [20].

The clinically relevant question is, if identified oncogenic signaling molecules and pathways that induce an acquired resistance to CDK4/6 inhibitors can be targeted to overcome this resistance. For this purpose, we extracted signaling pathways that are frequently mutated in bladder cancer patients and demonstrated that combinations with inhibitors targeting RTKs, PI3K-AKT and Ras/MAPK exhibited synergism with Palbociclib. Recently, resistance mechanisms to CDK4/6 inhibitors have been published by using different experimental approaches. For instance, a kinome-wide RNA interference, exome sequencing and a drug screen identified activation of the PI3K, JAK/STAT and MAPK pathway and modulators of histone modification as mechanisms of acquired resistance and inhibitors against different key molecules in these pathways in breast cancer, myeloproliferative neoplasia, mucosal melanomas and leukemia overcame this resistance [18, 19, 53–55]. In particular, RTK/PI3K-Akt pathway inhibitors and CDK2 inhibitors have been evaluated in combination with CDK4/6 inhibitors very detailed in other tumor entities before [18, 19, 56, 57]. These results are in accordance with the results obtained from our CRISPR-dCas9 approach and link activation of multiple pathways to resistance to CDK4/6 inhibition [20]. We also extend this knowledge by demonstrating that RTK inhibitors Axitinib and Erdafitinib showed synergism as combination therpay with Palbociclib. Given the complexity in targeting the PI3K signaling pathway in cancer [58], our results also demonstrate that a strategy to target mTOR and PI3K kinase by using a dual inhibitor results in better effects in combination with Palbociclib than the combination of other inhibitors in this pathway. However, unlike in myoproliferative neoplasms, the use of inhibitors against the JAK/STAT signaling pathway in combination with Palbociclib was ineffective in BLCA and needs further investigation [54].

Our results confirm and advocate that use of genome-scale CRISPR-dCas9 screening approaches are an extremely useful “all in one” strategy for the identification of predictive markers resulting in the identification of signaling pathways that can be used for combination therapy development.

A challenge in the design of clinical trials using CDK4/6 inhibitors is the molecular pre-stratification for mono- or combination therapy of patients according to their genetic background to achieve a maximum benefit [13–15]. Only RB1 expression has been regarded to date as the most important promising biomarker based on preclinical findings that RB negative cell lines do not respond to therapy in almost all publications,not. This observation is also supported in patients with acquired resistance to CDK4/6 inhibitors in whom novel mutations in RB1 with predicated loss of function have been observed [59]. However, results from our screen identified almost a thousand genes that potentially can serve as predictive markers. This could reflect the complexity by which CDK4/6 inhibitors regulate downstream events and also explain why the combination with inhibitors against multiple pathways can act synergistically [20]. As for improvement of patient stratification, we offer here a strategy in which one could stratify patients based on activation of distinct pathways and combine CDK4/6 inhibitors with inhibitors targeting those pathways.

## Conclusion

In conclusion, we could demonstrate that use of a CRISPR-dCas9 genome scale gain of function screen for elucidating mechanisms of resistance to target therapies does reveal a dataset that allows the design of suitable strategies for combination treatment. This experimental approach also enabled a better mechanistic understanding of biological processes that are involved in signaling events related to CDK4/6 inhibition and thus might also facilitate identification of a predictive marker panel that can be used in clinical practice.

## Additional files

Additional file 1 **Figure S1.** Characterization of T24 SAM clones and quality control of NGS data. (a) Expression of dCas9 and MS2-P65-HSF1 activation helper in T24 SAM clones. (b) Response of T24WT and T24 SAM2 cells to Palbociclib were evaluated by CTB assay. (c) Expression of urinary markers of key cell lines with MCF7 as control. (d) Cell viability assay of Zeocin (300μg/ml) on T24 SAM cells treated with different amounts of supernatant containing lentivirus for evaluating the MOI. NGS reads and counts were analyzed using MaGeck-VISPR for (e) sequence quality, (f) percentage of mapped reads, (g) Gini index, (h) count distribution, (i) percentage of missing gRNAs. (PDF 8899 kb)

Additional file 2: **Figure S2.** Non-linear regression dose-response curve with mono- and combination therapies on T24/RT112 cell lines. X-axis represents the concentration gradient of monotherapies or the combination with Palbociclib (1000 nM). Y-axis represent the effect on cell viability (Data from 3 independently biological replicates). (a) T24 cells (b) RT112 cells. (PDF 702 kb)

Additional file 3: **Figure S3.** Effects of combination therapies on cell cycle progression, caspase3/7 activity and long-term synergy measurement on RT112. (a) Cell cycle progression was analyzed after 72 h of treatment. (b) Caspase3/7 activity was measured and normalized to CTB results after 72 h of treatment (*, *P* < 0.05; one-way ANOVA with Dunnett’s multiple comparisons test and unpaired t-test; NS not significant). Data represent the mean ± SD of 3 replicates.). (c) Effects on proliferation after 7 days of treatment with Palbociclib were evaluated with SRB assay and quantified (*, P < 0.05; one-way ANOVA with Dunnett’s multiple comparisons test). Concentrations applied were Palbociclib (1000 nM) alone or in combination with Axitinib (1000 nM), Erdafitinib (5000 nM), NVP-BEZ235 (200 nM) and CI1040 (1000 nM). (PDF 3220 kb)

Additional file 4: **Figure S4.** Western blot analysis against molecules involved in therapy response in T24 cells (a, b, c, d) 3 days after treatment, cell lysates from control, monotherapies and combination therapies were analyzed by immunoblot with the indicated antibodies. (PDF 5997 kb)

Additional file 5: **Figure S5.** Western blot analysis against molecules involved in therapy response in RT112 cells (a, b, c, d) 3 days after treatment, cell lysates from control, monotherapies and combination therapies were analyzed by immunoblot with the indicated antibodies. (PDF 5748 kb)

Additional file 6: **Table S1.** Genotyping of cell lines used and list of primers and sgRNAs. (a) Genotyping of T24 and RT112, (b) List of primer and sgRNA sequences used. (XLSX 12 kb)

Additional file 7: **Table S2.** Analysis of screen and TCGA data. (a) NGS analysis with MaGeck (significant candidate sgRNAs with FDR cutoff 0.1 and high_in_treatment = true). (b) list of converted screen sgRNA candidates to official gene symbols, (c) Candidate genes with clinical amplification on CNA based on Comprehensive analysis of muscle-invasive bladder cancers characterized by multiple TCGA analytical platforms on CbioPortal. (XLSX 9460 kb)

Additional file 8: **Table S3.** IC50 and Combination Index analysis of combination therapies in RT112 cells. (XLSX 9 kb)

## Data Availability

All the NGS data generated and analyzed during this study can be found at Mendeley online library with DOI:10.17632/ptx3dzs926.1. Further details are available from the corresponding author upon request.

## Abbreviations

BLCA: Bladder Urothelial Carcinoma
CAM: Chicken Chorioallantoic Membrane
CDK4/6: Cyclin-Dpendent Kinase4/6
CI: Combination Index
CNA: Copy Number Alteration
CRISPR: Clustered Regularly Interspaced Short Palindromic Repeats
dCas9: dead CRISPR associated protein 9
ED: Embryo Day
FDR: False Discovery Rate
GOF: Gain of Function
IC50: half maximal Inhibitory Concentration
LFC: Log2 Fold Change
MOI: Multiplicity of Infection
NGS: Next-Generation Sequencing
NTC: Non-Target Control
PCR: Polymerase Chain Reaction
SAM: Synergistic Activation Mediator
SgRNA: Single-Guide RNA
SRB: Sulforhodamine B
