# Supplementary figures and images for: Functional genomics identifies predictive markers and clinically actionable resistance mechanisms to CDK4/6 inhibition in bladder cancer
Source: J Exp Clin Cancer Res. 2019 Jul 22;38:322. doi: 10.1186/s13046-019-1322-9 (PMC6647307; doi:10.1186/s13046-019-1322-9)

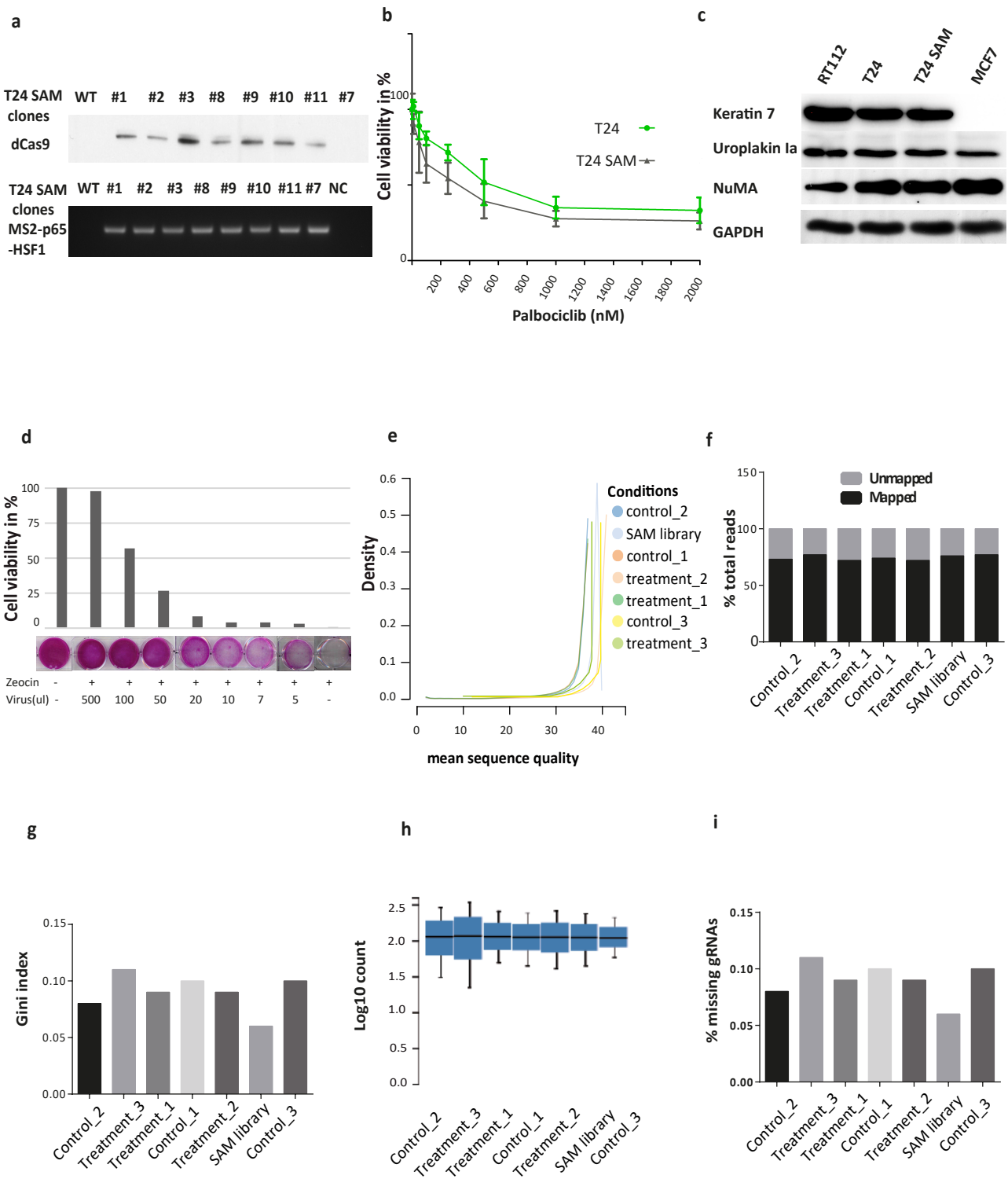

Supplement: Supplementary file 1 — Figure S1. Characterization of T24 SAM clones and quality control of NGS data. (a) Expression of dCas9 and MS2-P65-HSF1 activation helper in T24 SAM clones. (b) Response of T24WT and T24 SAM2 cells to Palbociclib were evaluated by CTB assay. (c) Expression of urinary markers of key cell lines with MCF7 as control. (d) Cell viability assay of Zeocin (300μg/ml) on T24 SAM cells treated with different amounts of supernatant containing lentivirus for evaluating the MOI. NGS reads and counts were analyzed using MaGeck-VISPR for (e) sequence quality, (f) percentage of mapped reads, (g) Gini index, (h) count distribution, (i) percentage of missing gRNAs. (PDF 8899 kb) [file 13046_2019_1322_MOESM1_ESM.pdf]

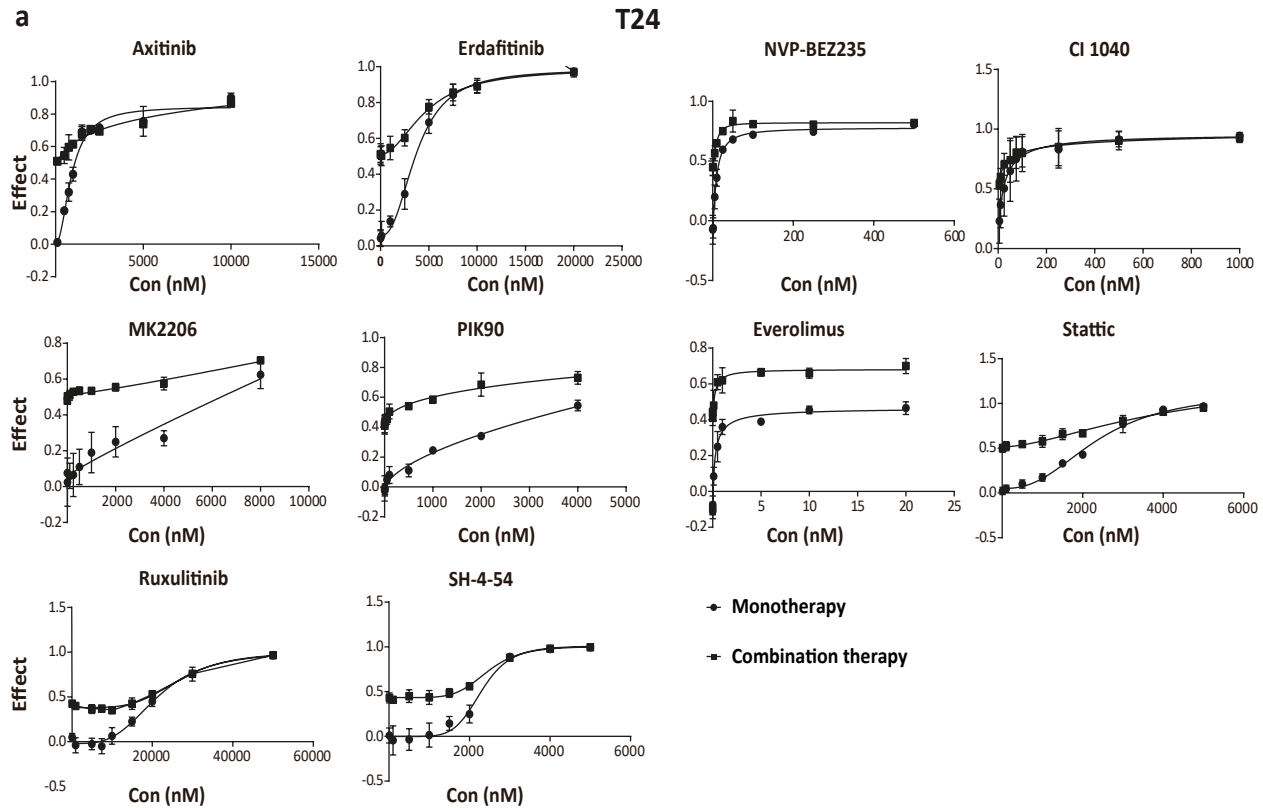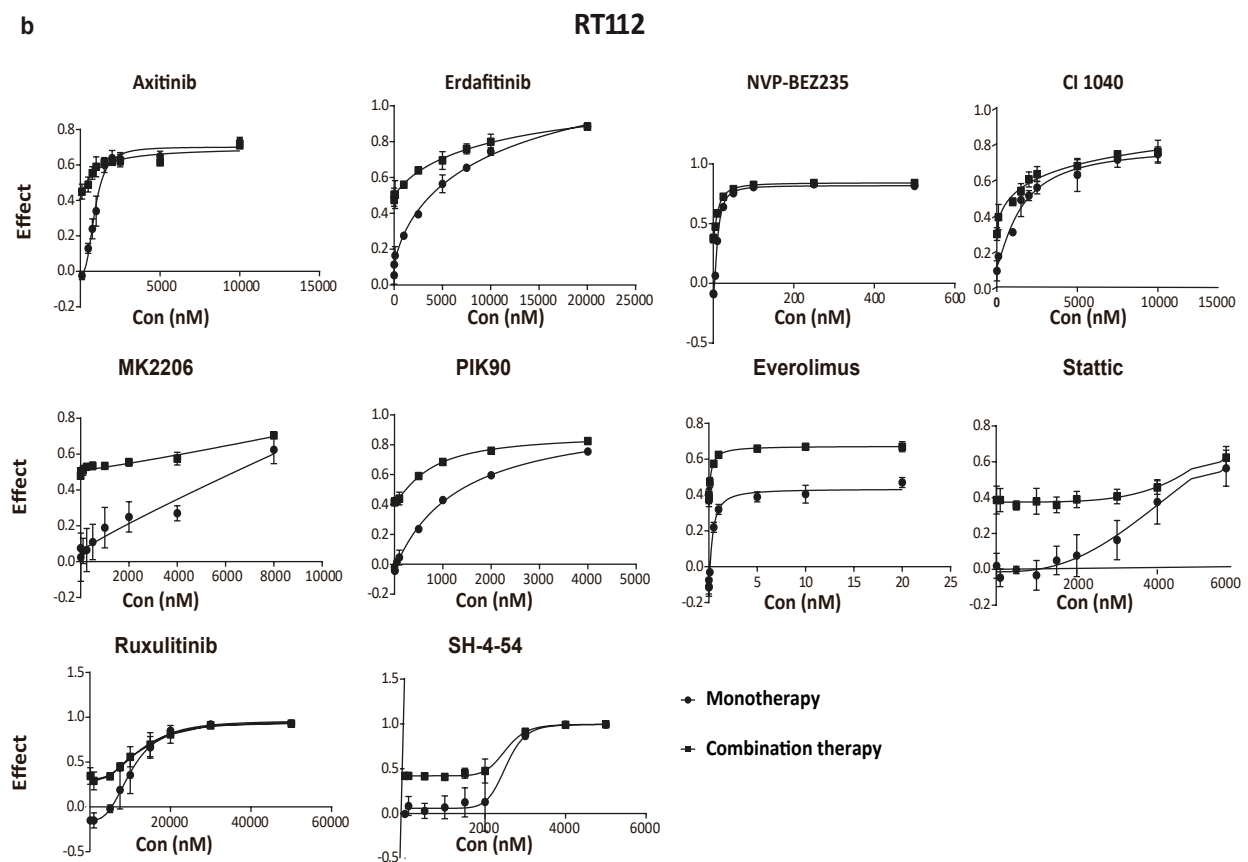

Supplement: Supplementary file 2 — Figure S2. Non-linear regression dose-response curve with mono- and combination therapies on T24/RT112 cell lines. X-axis represents the concentration gradient of monotherapies or the combination with Palbociclib (1000 nM). Y-axis represent the effect on cell viability (Data from 3 independently biological replicates). (a) T24 cells (b) RT112 cells. (PDF 702 kb) [file 13046_2019_1322_MOESM2_ESM.pdf]

**a**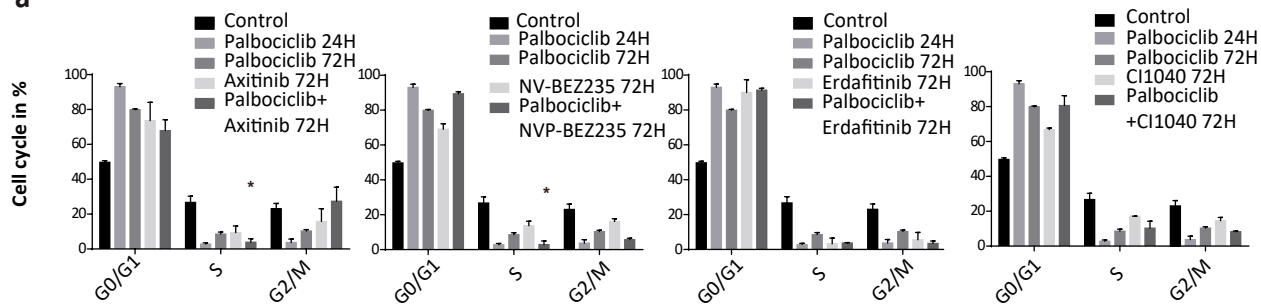**b**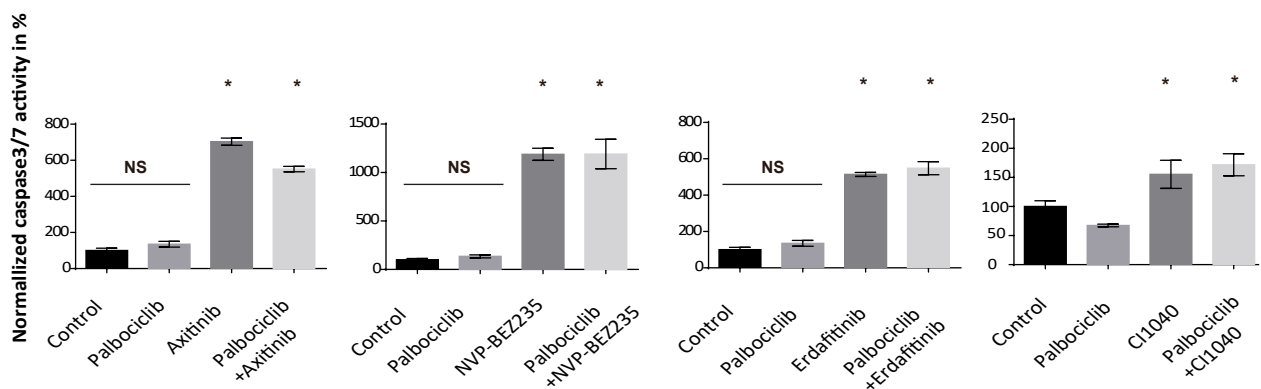**c**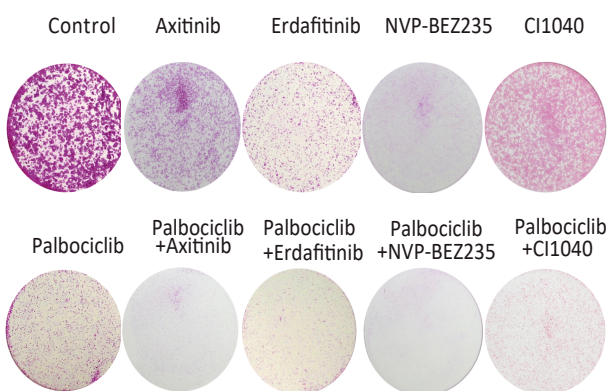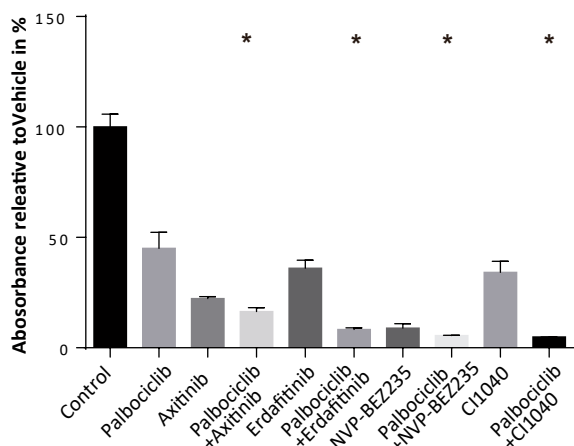

Supplement: Supplementary file 3 — Figure S3. Effects of combination therapies on cell cycle progression, caspase3/7 activity and long-term synergy measurement on RT112. (a) Cell cycle progression was analyzed after 72 h of treatment. (b) Caspase3/7 activity was measured and normalized to CTB results after 72 h of treatment (*, P < 0.05; one-way ANOVA with Dunnett’s multiple comparisons test and unpaired t-test; NS not significant). Data represent the mean ± SD of 3 replicates.). (c) Effects on proliferation after 7 days of treatment with Palbociclib were evaluated with SRB assay and quantified (*, P < 0.05; one-way ANOVA with Dunnett’s multiple comparisons test). Concentrations applied were Palbociclib (1000 nM) alone or in combination with Axitinib (1000 nM), Erdafitinib (5000 nM), NVP-BEZ235 (200 nM) and CI1040 (1000 nM). (PDF 3220 kb) [file 13046_2019_1322_MOESM3_ESM.pdf]

**a**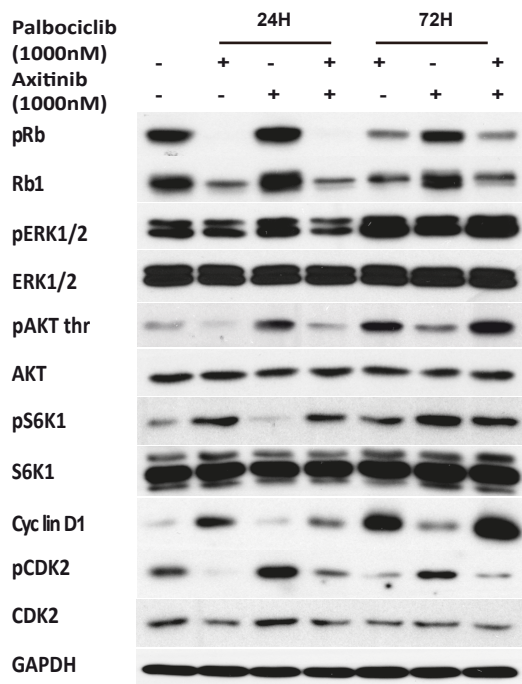**c**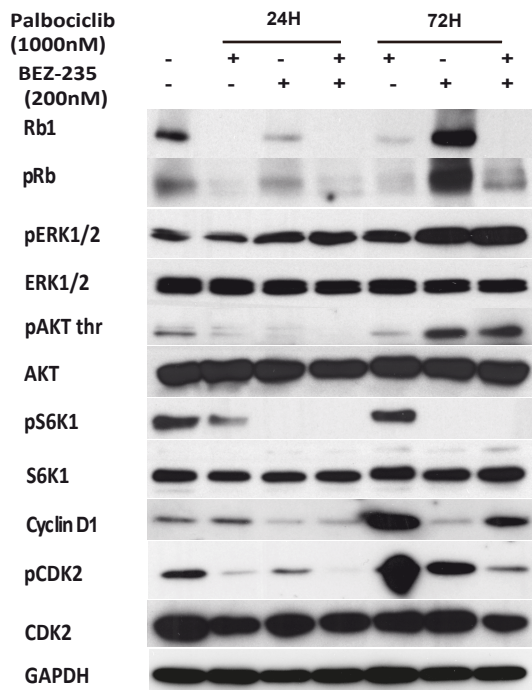**b**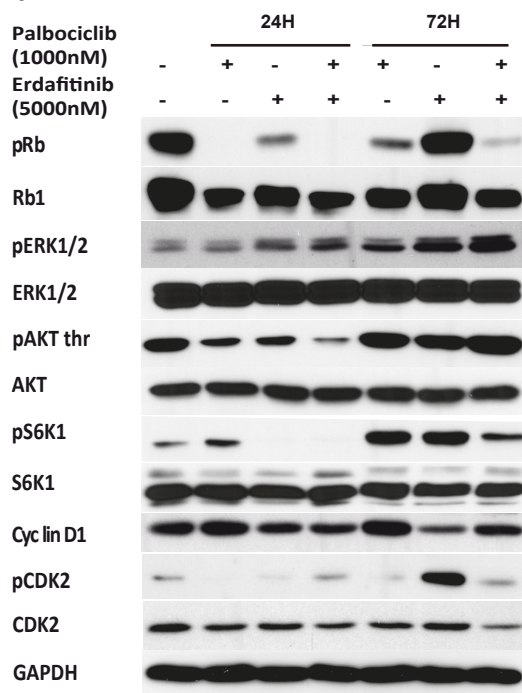**d**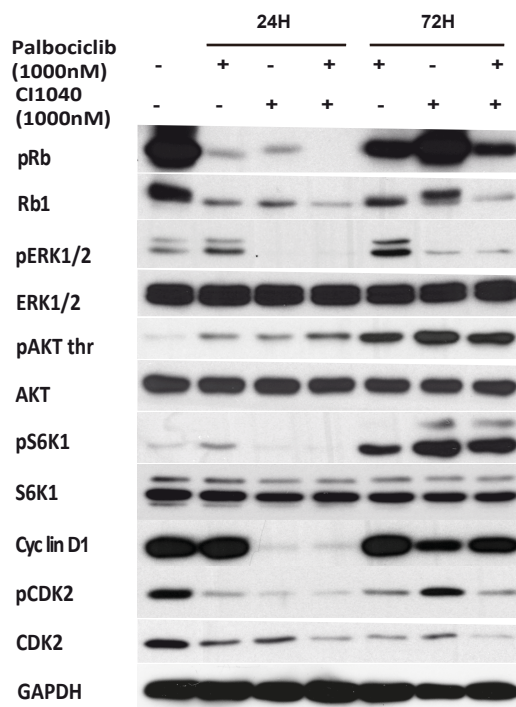

Supplement: Supplementary file 4 — Figure S4. Western blot analysis against molecules involved in therapy response in T24 cells (a, b, c, d) 3 days after treatment, cell lysates from control, monotherapies and combination therapies were analyzed by immunoblot with the indicated antibodies. (PDF 5997 kb) [file 13046_2019_1322_MOESM4_ESM.pdf]

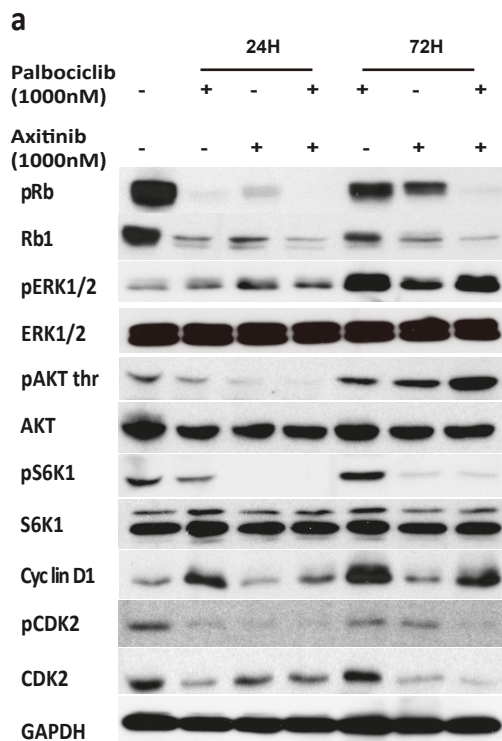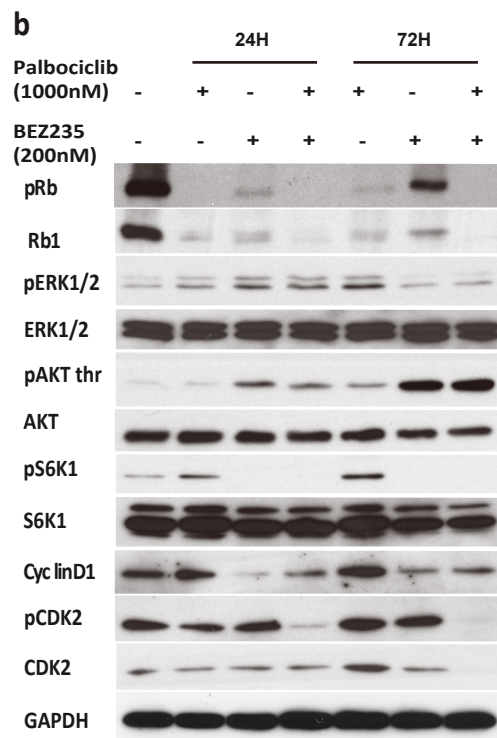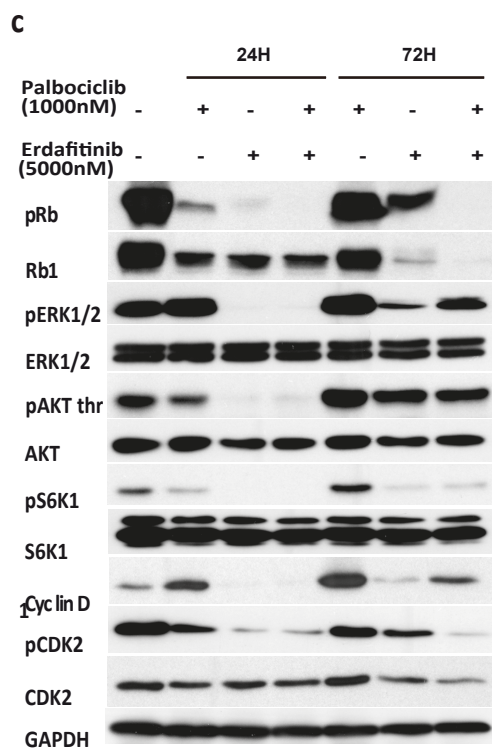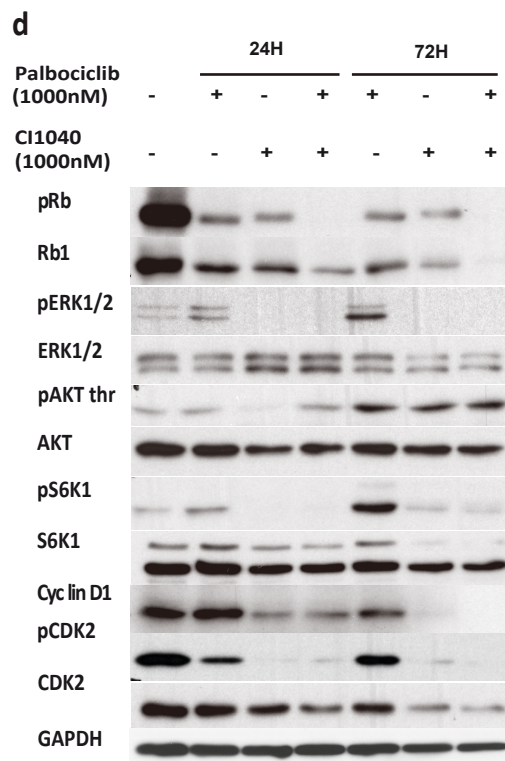

Supplement: Supplementary file 5 — Figure S5. Western blot analysis against molecules involved in therapy response in RT112 cells (a, b, c, d) 3 days after treatment, cell lysates from control, monotherapies and combination therapies were analyzed by immunoblot with the indicated antibodies. (PDF 5748 kb) [file 13046_2019_1322_MOESM5_ESM.pdf]
